# Evaluation of an artificial intelligent hydrocephalus diagnosis model based on transfer learning

**DOI:** 10.1097/MD.0000000000021229

**Published:** 2020-07-17

**Authors:** Weike Duan, Jinsen Zhang, Liang Zhang, Zongsong Lin, Yuhang Chen, Xiaowei Hao, Yixin Wang, Hongri Zhang

**Affiliations:** a Department of Neurosurgery, the First Affiliated Hospital of Henan University of Science and Technology, Luoyang; b Department of Neurosurgery, Huashan Hospital, Fudan University; c Shanghai Nanoperception Information Technology Co. Ltd, Shanghai, P.R. China; d Vaccine and Infectious Disease Division, Fred Hutchinson Cancer Research Center, Seattle, USA.

**Keywords:** artificial intelligent, computer tomography, hydrocephalus, transfer learning

## Abstract

To design and develop artificial intelligence (AI) hydrocephalus (HYC) imaging diagnostic model using a transfer learning algorithm and evaluate its application in the diagnosis of HYC by non-contrast material-enhanced head computed tomographic (CT) images.

A training and validation dataset of non-contrast material-enhanced head CT examinations that comprised of 1000 patients with HYC and 1000 normal people with no HYC accumulating to 28,500 images. Images were pre-processed, and the feature variables were labeled. The feature variables were extracted by the neural network for transfer learning. AI algorithm performance was tested on a separate dataset containing 250 examinations of HYC and 250 of normal. Resident, attending and consultant in the department of radiology were also tested with the test sets, their results were compared with the AI model.

Final model performance for HYC showed 93.6% sensitivity (95% confidence interval: 77%, 97%) and 94.4% specificity (95% confidence interval: 79%, 98%), with area under the characteristic curve of 0.93. Accuracy rate of model, resident, attending, and consultant were 94.0%, 93.4%, 95.6%, and 97.0%.

AI can effectively identify the characteristics of HYC from CT images of the brain and automatically analyze the images. In the future, AI can provide auxiliary diagnosis of image results and reduce the burden on junior doctors.

## 1 Introduction

Hydrocephalus (HYC) is a common disorder in neurosurgery. Non-contrast material-enhanced head computed tomographic (CT) examination is an important method for the diagnosis of HYC because it can observe the enlargement of the ventricles, and sometimes determine the cause of HYC.^[1,2]^ However, due to the lack of uniform standards, different range of patients’ ages and the various levels doctors’ expertise, it is rather difficult to reach a diagnosis. Therefore, using new technologies to explore diagnostic methods and standards has great value for HYC. With the development of artificial intelligence (AI), deep learning has achieved in many medical diagnoses field.^[3–5]^ However, it is difficult to obtain a large amount of medical image data to train an AI model. One method of addressing this lack of data in a given domain is to transfer the learned model parameters to the new model, a technique known as transfer learning. Transfer learning has proven to be a highly effective technique, particularly when faced with domains with limited data.^[6]^ Therefore, the purpose of this study was to develop an AI diagnostic model for HYC using CT images on the basis of transfer learning and evaluate its performance to detect HYC within a range of non-contrast-enhanced head CT examinations, thereby performing an initial assessment to assist radiologists

## 2 Materials and methods

### 2.1 Data collection

The study protocol was approved by the Ethics Committee of the First Affiliated Hospital of Henan University of Science and Technology (Luoyang, Henan, China).

CT examination was performed in 16-slice spiral CT scanner (Phillips, The Netherlands). Axial sections were obtained at 6-mm slice thickness from the skull base to the vertex along the window center 40 HU and width 90 HU.

The diagnostic index of HYC is Evan index, Evan index ≤0.32 is normal, > 0.32 is diagnosed as HYC.^[7]^ Three radiologists read every examination and got diagnosis. When results of the 3 radiologists were the same, the diagnosis was established and the subject was included in the study. One thousand two hundred fifty examinations of HYC patients (685 men and 565 women; mean age: 53.26 ± 19.11; age range: 14–89 years) in the First Affiliated Hospital of Henan University of Science and Technology from June 2012 to June 2018 were collected. One thousand two hundred fifty examinations of normal people with no HYC were collected matched to the age and sex of HYC patients from March 2015 to June 2018. The ratio of HYC patients to normal people are1:1. There were no difference in age and sex between HYC patients and normal people. Ten to twenty layers of each CT examination (upward includes all lateral ventricles and downward includes the eye scan layer) were extracted for analysis. For these 1250 subjects, we randomly upset their order and further divided them into 3 parts: training (60%), validation (20%), and test (20%). Each part of the dataset is independent of each other, thus avoiding the training dataset is applied to the process of testing.

### 2.2 Pre-processing of images and tagging of feature variables

Python is an interpreted, high-level, general-purpose programming language. In this study, python was selected to develop marking tool. Different colors were used to mark brain structure including lateral ventricle, third ventricle, aqueduct, fourth ventricle, lateral fissure. The marking work of those images was completed by 3 residents of radiology and confirmed by a consultant of radiology.

The pre-processing of images in our study consists of 3 parts: segmentation, building input datasets, and data augmentation. After marked, CT images can be further segmented into HYC ventricular system, normal ventricular system, and brain tissue regions. Input datasets include all images which had been marked. Through data augmentation, performing some pre-processing on the original data can speed up network convergence and improve accuracy. The details of the data augmentation methods implemented in this study are as follows:

(1) Flip the picture up and down, left and right randomly;
(2) Rotating the slice between 10 degrees randomly;
(3) Shifting the slice between 15 pixels randomly.

Each slice of the network input needed to be carried out using the same rotating/shifting operation in 1 augmentation.

### 2.3 Network architecture

DenseNet encourages feature reuse and reduces the number of parameters, which not only lowers the requirements on the hardware device but also has the benefit of good feature extraction. Based on this, in this study DenseNet was conducted to extract features for HYC estimation. Then we carry out further fine-tuning towards the neural network results and network parameters to improve the accuracy of the algorithm. After that, the training model and validation set were used to train the algorithm model.

To speed up the training, batch normalization was used. After batch normalizing transform, the sample xi (a mini-batch of size n) have been normalized into yi, as shown in Table 1. Moreover, in this transform, ϵis a constant to ensure the stability of 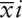. To prevent over fitting, a dropout rate of 0.5 was applied to the fine-tuning of our network.

**Table 1:**
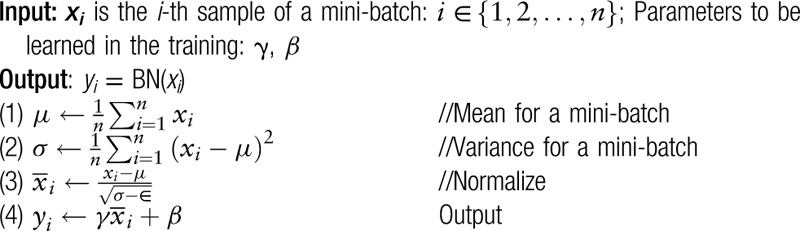
Batch normalizing transform.

Loss function plays an important role in the process of training the model. Mean absolute error (MAE) loss and mean square error (MSE) loss, as 2 different types of loss functions, are widely used to solve regression problems. Compared with MSE, MAE can better reflect the actual. In the process of training the model, MAE was selected as the loss function, which was defined as follows:
 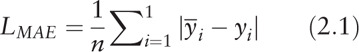


The parameters related to HYC ventricular volume, cerebrospinal fluid volume, cranial cavity volume, maximum length of frontal angle of lateral ventricle, maximum width of brain and Evan index were input to neural network to improve the accuracy of the algorithm.

### 2.4 Testing process of model and radiologist

In the process of testing radiologists, 2 residents, 2 attendants, and 2 consultants were chosen to read CT images and required to have a diagnosis. All of physicians are from Imaging Medical Center of the First Affiliated Hospital of Henan University of Science and Technology. CT image data was converted to JPG format (window width, 90 HU; window center, 40 HU) for reading by each radiologist.

In our study, MAE and root MSE were chosen as evaluation metrics, which applied to determine whether the model can solve the problem well, which are defined as follows:
 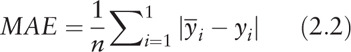 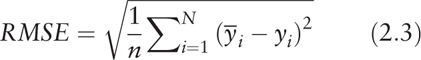


### 2.5 Statistical analysis

SPSS ver. 19.0 software (SPSS, Inc., Chicago, IL) was used for statistical analysis. All data were presented as the mean ± standard deviation.

## 3 Results

**Figure 1:**
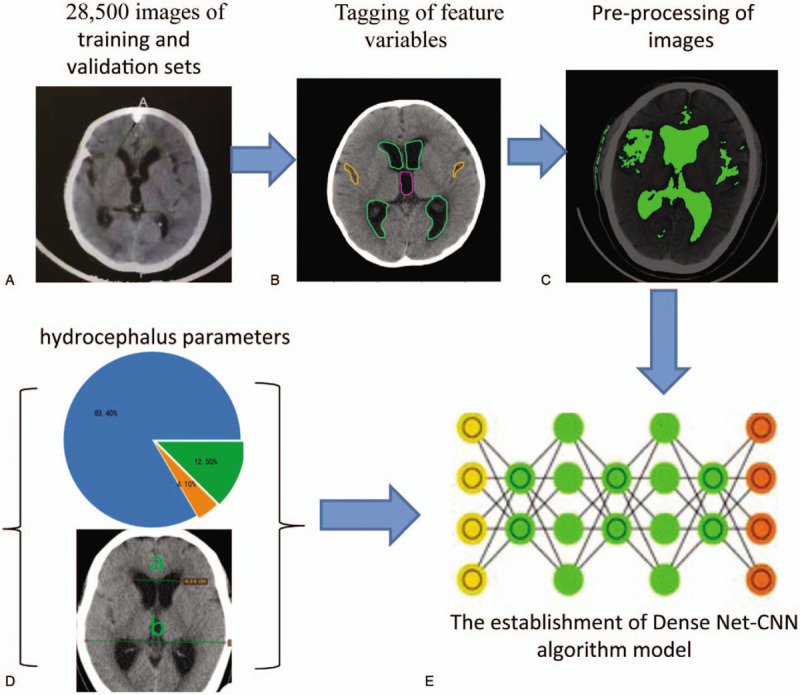
Work flow of establishing artificial intelligence hydrocephalus diagnosis model.

A tool that can read DICOM data had been developed (Fig. 1A), and radiologists can use it to tag the feature variables of the images (Fig. 1B). This tool can also automatically identify cerebrospinal fluid and brain tissue (Fig. 1C). Combining with the labeled feature variables and parameters related to HYC, Evan index were input to neural network (Fig. 1D). AI model was developed through machine learning (Fig. 1E).

**Figure 2:**
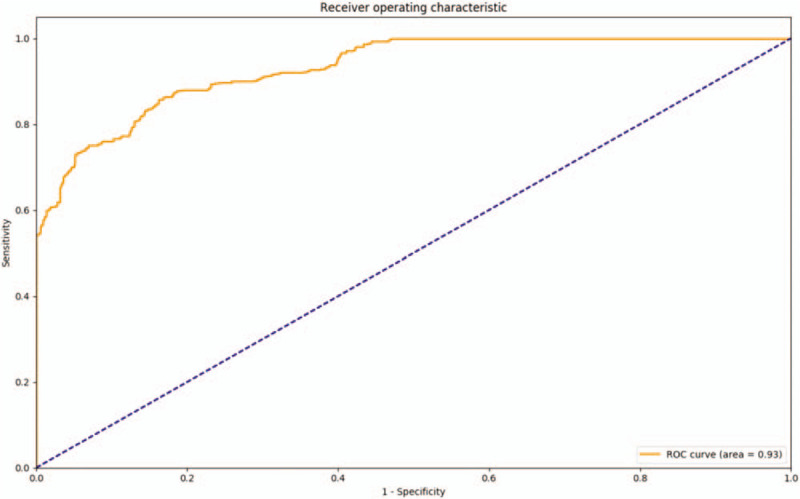
The ROC curve of the model. The area under the ROC curve was 0.93.

This study indicates that the AI diagnosis model can diagnose hydrocephalus by reading brain CT images. It achieves this function by analyzing the factors of the shape and volume of ventricle, Evan index and age, which is a new method for diagnosing hydrocephalus. The final algorithm performance of model shows an accurate rate of 94.0% (Table 2), a specificity of 94.4% (95% CI: 79%, 98%) with a sensitivity of 93.6% (95% CI: 77%, 97%) and the area under curve of 0.93 (Fig. 2).

**Table 2:**
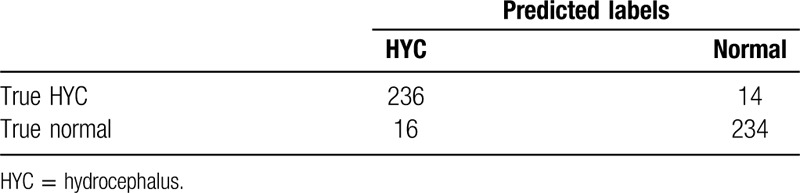
Result of model test.

**Figure 3:**
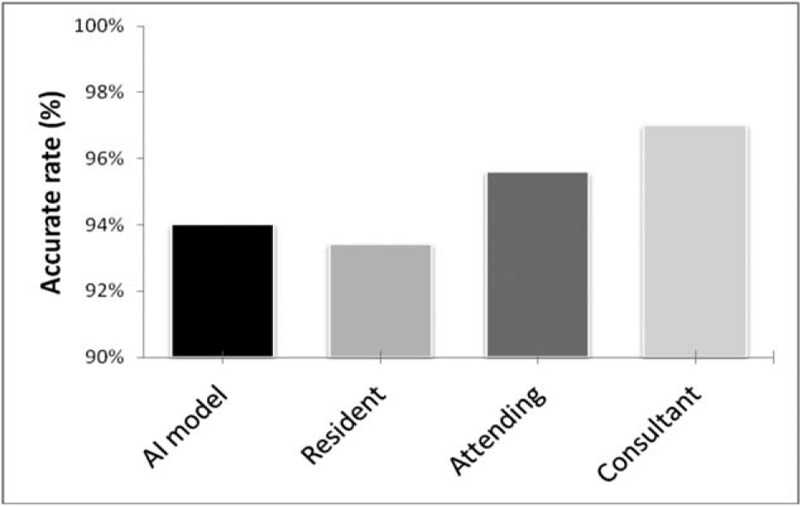
Multi-class comparison between model and physicians. The diagnostic accuracy of artificial intelligence model is comparable to that of resident and lower than that of attending and consultant.

The resident with the accurate rate of 93.4% (Table 3), the attending accurate rate 95.6% (Table 4), and consultant 97.0% (Table 5) was shown. The results showed that the diagnostic capabilities of AI model are comparable to those of junior doctors, with high performance in terms of accuracy, sensitivity, specificity, and precise diagnosis. However, there are still differences in comparison with senior doctors (Fig. 3).

**Table 3:**
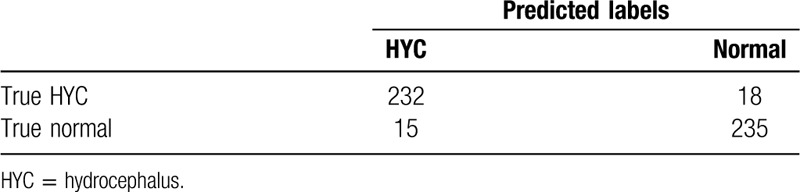
Result of resident physicians test.

**Table 4:**
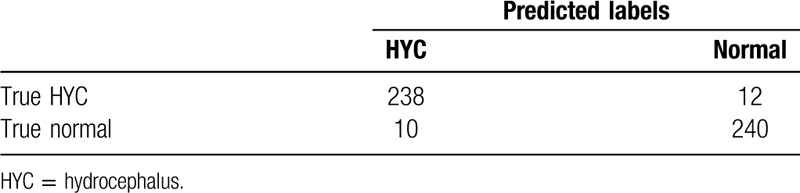
Result of attending physicians test.

**Table 5:**
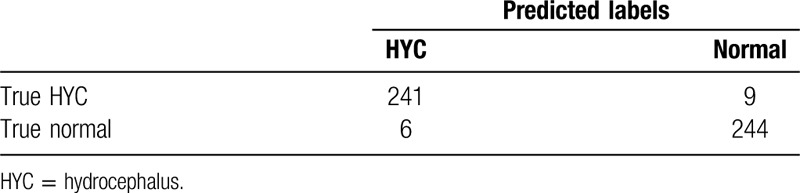
Result of deputy chief physicians test.

## 4 Discussion

This study used 35,500 images obtained from 2500 CT examination to train an AI model for HYC diagnose. The AI model achieved good performance using a transfer learning algorithm. Compared with classical deep learning, transfer learning can obtain a highly accurate model from a small training data set, although its performance is still less than that of classical deep learning using millions of data.^[8,9]^ In addition, classical deep learning usually takes more time to achieve the best accuracy than transfer learning. Because it is difficult to collect millions medical image datasets, this study chose transfer learning to train with CT head images.

The performance of transfer learning model depends to a large extent on that of the pre-training model.^[10,11]^ The performance of the transfer learning model will improve, if much more advanced learning techniques and involve more medical image datasets is used in pre-trained models. In addition, the rapid development of convolutional neural networks outside medical imaging will also provide better performance and training models for transfer learning.

Loss function was used in the process of training the model. MAE loss and MSE loss were used to solve regression problems such as age prediction. MAE can better reflect the actual situation of prediction error. MAE can also perform better than MSE in related HYC prediction problems.^[12]^ As a result, our study selected MAE as the loss function to predict brain age.

The commonly used index of ventriculomegaly includes the Evan index and the frontal-occipital angle ratio. The diagnosis of HYC not only involves a certain expansion of the ventricle, but also must be differentiated from other diseases, including Alzheimer disease and brain atrophy.^[13,14]^ The Evan index was mainly used to diagnose HYC due to the small sample size, thus the specificity of the model might increase with growing sample size. As the number of feature variables increases, this transfer learning model is expected to be able to diagnose diseases including Alzheimer disease and brain atrophy.

Prevedello et al^[15]^ recently reported that the accuracy rate of an AI model they developed for HYC diagnosis was up to 90%. The accuracy rate in this study was higher than their reported because of differences in the number of algorithms and data points. Note, however, that it is impossible to determine the pros and cons of a model solely by using the accuracy rate. Certain conditions are normally misdiagnosed as HYC, whereas further examination could exclude HYC. Early diagnosis of a patient with HYC is benefit to efficacy of treatment and prevent secondary impairment although HYC cause physical damage.

The format of CT examination in this study is DICOM, so our model can only recognize DICOM data. Future research can be extended to data in other formats, including JPG and FlashPix. Data can also be sourced from magnetic resonance images, X-rays, and digital subtraction angiography, making this type of AI model more practical and widely available. Meanwhile, such models can be applied to other diseases, including cerebral hemorrhage, cerebral infarction, and brain trauma, and can even be further extended to other disciplines. In view of the important guiding role of medical imaging in treatment, the application of AI to medical imaging diagnosis for evaluating disease, adjuvant therapy, and prognosis is a promising field for future research.^[16–18]^

Although scientific researchers are increasingly enthusiastic about AI, in fact AI is still in its “infancy” in the medical field.^[19–23]^ All studies are limited to verifying the feasibility or validity of AI technology.^[24]^ The application of AI in clinical practice could be quite popular in the future.

## Author contributions

**Investigation:** Jinsen Zhang.

**Methodology:** Jinsen Zhang.

**Writing – original draft:** Jinsen Zhang.
